# RNA Interference (RNAi) as a Potential Tool for Control of Mycotoxin Contamination in Crop Plants: Concepts and Considerations

**DOI:** 10.3389/fpls.2017.00200

**Published:** 2017-02-14

**Authors:** Rajtilak Majumdar, Kanniah Rajasekaran, Jeffrey W. Cary

**Affiliations:** Food and Feed Safety Research Unit, United States Department of Agriculture – Agricultural Research Service, Southern Regional Research Center, New OrleansLA, USA

**Keywords:** gene silencing, mycotoxin, fungi, disease resistance, host-induced gene silencing (HIGS), biotechnology, host–pathogen interaction, RNAi

## Abstract

Mycotoxin contamination in food and feed crops is a major concern worldwide. Fungal pathogens of the genera *Aspergillus. Fusarium*, and *Penicillium* are a major threat to food and feed crops due to production of mycotoxins such as aflatoxins, 4-deoxynivalenol, patulin, and numerous other toxic secondary metabolites that substantially reduce the value of the crop. While host resistance genes are frequently used to introgress disease resistance into elite germplasm, either through traditional breeding or transgenic approaches, such resistance is often compromised by the evolving pathogen over time. RNAi-based host-induced gene silencing of key genes required by the pathogen for optimal growth, virulence and/or toxin production, can serve as an alternative, pre-harvest approach for disease control. RNAi represents a robust and efficient tool that can be used in a highly targeted, tissue specific manner to combat mycotoxigenic fungi infecting crop plants. Successful transgenic RNAi implementation depends on several factors including (1) designing vectors to produce double-stranded RNAs (dsRNAs) that will generate small interfering RNA (siRNA) species for optimal gene silencing and reduced potential for off-target effects; (2) availability of ample target siRNAs at the infection site; (3) efficient uptake of siRNAs by the fungus; (4) siRNA half-life and (5) amplification of the silencing effect. This review provides a critical and comprehensive evaluation of the published literature on the use of RNAi-based approaches to control mycotoxin contamination in crop plants. It also examines experimental strategies used to better understand the mode of action of RNAi with the aim of eliminating mycotoxin contamination, thereby improving food and feed safety.

## Introduction

Mycotoxin contamination in food and feed crops, both pre- and post-harvest by phytopathogenic fungi is a major concern worldwide (Ismaiel and Papenbrock, 2015). Exposure to mycotoxins in humans and livestock occurs mainly through ingestion of contaminated seeds or other edible plant parts. The economic impact of mycotoxins is estimated to be $0.5–1.5 billion/year in the USA and Canada^1^. Aflatoxin contamination of maize costs producers approximately $163 million/year in the USA (Wu, 2006). Based on climate change predictions, it has been estimated that aflatoxin contamination could cause losses to the corn industry ranging from US$52.1 million to US$1.68 billion annually in the USA (Mitchell et al., 2016). There are also adverse health implications to humans and animals that consume aflatoxin contaminated foods and feeds. In the USA alone, the total annual losses due to the three major mycotoxins – aflatoxin, fumonisin, and deoxynivalenol are estimated to be as high as US$1 billion (Vardon et al., 2003). Based on the recent proposal to set maximum limits of ochratoxin A in food by the Canadian Health Department, Canadian food producers alone could experience estimated annual losses over 260 million Canadian dollars (CD) and the USA could suffer over 17 million CD in losses on food export to Canada (Wu et al., 2014).

Mycotoxin contamination can be both a pre- and post-harvest concern. In general mycotoxigenic fungi are present in the crop prior to storage. The majority of the mycotoxins produced in plants can be attributed mainly to the three fungal genera, *Aspergillus. Fusarium*, and *Penicillium*. The predominant mycotoxins produced by these necrotrophic fungi are often found in cereals and include aflatoxins, deoxynivalenol, fumonisins, fusarin C, fusaric acid, zearalenone, citrinin, patulin, penicillic acid, and ochratoxin A (Ismaiel and Papenbrock, 2015).

Besides conventional breeding approaches to introduce disease resistance traits into elite germplasm, more sophisticated biotechnological approaches are also being employed in the ongoing battle to control mycotoxigenic fungi. These include transgenic techniques that utilize RNA interference (RNAi), microRNA (miRNA)- or artificial microRNA (amiRNA)-mediated gene silencing, and designer transcription activator-like effector (dTALE)-mediated up or down-regulation of gene expression, to name a few (Bogdanove, 2014; Koch and Kogel, 2014; Tiwari et al., 2014). In addition, modern genome editing tools, e.g., Zn-Finger nucleases, mega-nucleases, transcription activator-like effector nucleases (TALEN), clustered regularly interspaced short palindromic repeats (CRISPR)/Cas9, and oligonucleotide-directed mutagenesis (ODN)-based gene editing techniques can be used to create mutations within the plant genome for trait improvement (reviewed by Sauer et al., 2016). Targeted genome editing, either to create a mutation within the existing genome or to add a gene(s) at a precise location in the genome, is mainly aimed toward genome alteration and associated trait development in the host plant. However, if the objective is to down-regulate the expression of key fungal pathogen genes that are required for disease progression in the host, then host induced gene silencing (HIGS) through RNAi might be the most robust tool to achieve such an objective. RNAi can be used in an inducible fashion to regulate gene expression in a spatio-temporal manner depending on the promoter used to drive the RNAi expression cassette. As RNAi negatively regulates gene expression at the post-transcription level and does not produce any terminal protein/enzyme in the host plant, this technology might have a greater acceptance to a broader audience if designed carefully to eliminate any off-target effects (discussed below).

RNA interference is a form of HIGS that is evolutionarily conserved in eukaryotes, the mechanism of which was first elucidated by Fire et al. (1998). Since its discovery, this natural phenomenon has emerged as a powerful tool for gene silencing and has been used extensively to help determine host gene function and create or improve existing plant traits associated with quantitative/qualitative yield attributes and stress tolerance (Kamthan et al., 2015). Besides manipulating host genes, RNAi technology has been successfully used to target genes of invading pathogens or pests that are critical for virulence and disease progression, and toxin production in the case of toxigenic plant pathogens (Sharma et al., 2013; Arias et al., 2015; Cheng et al., 2015). We will not be presenting detailed information about the mechanisms of RNAi in this review as this topic has been thoroughly reviewed by other authors (Tiwari et al., 2014; Chaloner et al., 2016). This review critically examines various aspects of RNAi technology that should be considered when developing control approaches. We also identify gaps in the knowledge that need to be addressed as well as providing examples of the application of RNAi for control of toxigenic fungi in crop plants.

## RNAi Pathway in Eukaryotes

**FIGURE 1:**
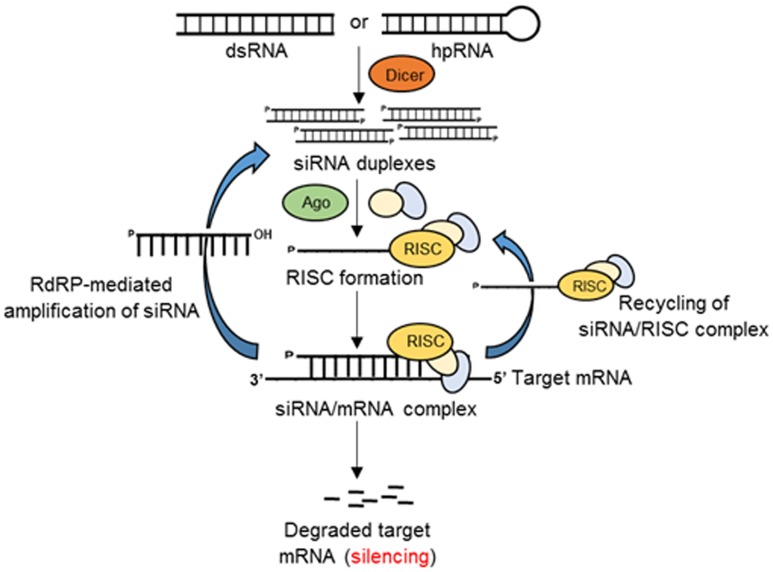
**Schematic of RNAi-mediated gene silencing in eukaryotes.** Double-stranded RNAs or hairpin RNAs (hpRNAs) generate small siRNA duplexes by the action of Dicer. The guide RNA strand binds with Argonaute (Ago) and other proteins to form an RNA-induced silencing complex (RISC). The siRNA/RISC complex then binds the complementary sequence of the target mRNA resulting in the degradation of the target transcript or inhibition of translation. The components of siRNA/mRNA complex can be recycled to the RISC complex or generate siRNA duplexes by the action of RNA-dependent RNA-polymerase (RdRP).

RNAi in eukaryotes (**Figure 1**) is an RNA-dependent gene silencing process, which is initiated by a RNAse III enzyme (Dicer) that cleaves a long double-stranded RNA (dsRNA) into double stranded small (∼20–25 bp nucleotide) interfering RNAs (siRNAs) with a two-nucleotide overhang at the 3′ end. Each siRNA is composed of a passenger (sense) strand and a guide (antisense) strand. While the guide strand is incorporated into an active RNA-induced silencing complex (RISC), the passenger strand is degraded by subsequent cellular events in the cytoplasm. The guide strand of the siRNA–RISC complex then base-pairs with the complementary mRNA target sequences and initiates endonucleolytic cleavage through the action of induced Argonaute protein (AGO; catalytic component of the RISC complex), thus preventing translation of the target transcript (Borges and Martienssen, 2015).

## Components of RNAi Machinery in Pathogen Virulence and Host Resistance or Susceptbility

### RNAi Machinery in Fungi: Impact on Growth and Pathogenicity

Different components of the fungal RNAi machinery are reported not only to play a role in fungal growth and development but also for pathogenesis. Double knockout mutants of the two Dicer (*Dcl*) genes in *Botrytis cinerea* show reduced virulence due to the lack of plant immune-suppressing *B. cinerea* siRNAs produced by the *Dcl* genes (Weiberg et al., 2013). In the plant pathogenic fungus *Colletotrichum higginsianum. Δdcl1. Δdcl1Δdcl2* double mutant, and *Δago1* strains exhibited abnormalities in conidiation and conidia morphology (Campo et al., 2016). Small RNA deep-sequencing and follow up experiments revealed that reduced expression of a dsRNA mycovirus [termed *Colletotrichum higginsianum non-segmented dsRNA virus 1* (*ChNRV1*)] in the above mutants was the cause of defective conidia development. In another study by Wang et al. (2016), using transgenic *Arabidopsis* and tomato plants overexpressing hpRNAs or exogenous application of synthetic hpRNAs to a wide variety of plants (tomato, strawberry, grapes, lettuce, onion, and rose) to dual silence *Bc-Dcl1* and *Bc-Dcl2* genes, resulted in significant reduction of fungal pathogenicity and growth. These findings signify the cross-kingdom movement of sRNAs from plants to fungi and the role of *Dcl* genes in fungal virulence, thereby suggesting *Dcl* genes as promising targets to control fungal growth and pathogenicity through RNAi-based approaches in a broad range of plants.

### Interaction of Fungal sRNAs with the Host RNAi Machinery and Silencing of Host Defense Genes

Fungal sRNAs can interact with host RNAi machinery to down-regulate host defense genes and enhance pathogenicity. It will be important to know the origin and diversity of these sRNAs in fungi and their corresponding host targets. Fungal sRNAs, transported through vesicles, have been shown to down-regulate host genes upon entering into the host cells. In fact, these sRNAs can take advantage of the host RNAi machinery to silence host genes primarily associated with defense pathways. In tomato and *Arabidopsis*, sRNAs secreted by the fungus *B. cinerea*, utilize plant *Ago1* to selectively silence host defense genes, namely mitogen-activated protein kinases (*MAPK*s), oxidative stress-related gene *peroxiredoxin* (*PRXIIF*), and cell wall-associated kinase (*WAK*) (Weiberg et al., 2013). An *Arabidopsis ago1* mutant showed reduced susceptibility to *B. cinerea*, whereas a *dcl1dcl2* double mutant of *B. cinerea*, incapable of producing these sRNAs, exhibited reduced pathogenicity. Retrotransposon-derived siRNAs in *B. cinerea* even with 3–5 bp mismatch could still effectively silence host defense genes, suggesting flexibility of the fungi to overcome host defenses (Weiberg et al., 2013). As more is learned about the identity and roles of fungal sRNAs in down-regulation of host defense genes, approaches for control of toxigenic fungal pathogens can be developed based on targeting of the genes encoding these sRNAs by host plant-based RNAi.

In rice (*Oryza sativa* L.), silencing of the *OsDCL1* gene showed enhanced resistance to the rice blast pathogen, *Magnaporthe oryzae*, in a non-race specific manner as well as constitutively activating other defense genes (Zhang et al., 2015). There appears to be a pathogen-specific interaction with the plant RNAi components, as it is not always true that mutation of genes associated with plant RNAi machinery will increase pathogen resistance. For example, *Arabidopsis AGO1* and *AGO2* mutants showed increased susceptibility to the necrotrophic fungus *Sclerotinia sclerotiorum* while over-expression of *AGO1* increased resistance to *S. sclerotiorum* in an expression dependent manner (Cao et al., 2016). Proper knowledge of host RNAi silencing components that can affect the degree of pathogenicity of the invading fungus, could be used to develop RNAi-based vectors targeting both host and fungal genes (through an inducible RNAi system) to restrict fungal growth and toxin production during infection.

## Specificity of RNAi

Without question the major concern of RNAi-based genetically engineered (GE) plants is the risks associated with off-target effects (Casacuberta et al., 2015; Roberts et al., 2015). An “off-target” effect (OTE) refers to any gene being silenced that is not the intended target, either in the organism producing the dsRNA or in an organism exposed to the dsRNA that is not the intended target organism (Roberts et al., 2015). Initial studies of gene silencing by siRNA suggested that the process was highly specific and just one base mismatch could abolish silencing. This was soon debunked by studies that demonstrated sequence-specific, silencing of off-target genes (Jackson et al., 2003; Persengiev et al., 2004; Jackson et al., 2006). The unintended target genes were reported to share partial sequence complementarity with the siRNA guide strand (Jackson et al., 2006). Off-target effects were reported to occur with as few as 15 out of 19 base pairs of complementarity between the siRNA and the target (Jackson et al., 2003).

Whether one is designing a single siRNA or an RNAi hairpin construct capable of producing a number of siRNAs specific for the target gene, it is critical that the siRNA(s) targeting the mRNA will have a high efficiency of silencing as well as a low probability of binding to off-target mRNAs. The most practical means to identify sequence complementarity between the expressed siRNAs and all known off-target mRNAs of the host plant and non-target organisms (NTOs) is to perform genome-wide bioinformatics analyses of all deposited transcriptome sequence data. This is usually accomplished with algorithms such as NCBI’s blast program or other web-based applications that can search and identify potential off-target genes from other organisms (reviewed in Li and Cha, 2007). Unfortunately, the usefulness of the NCBI blastn algorithm is hindered somewhat by its inability to accurately predict local alignments of short sequences. Using gene expression profiling of human cells transfected with 12 siRNAs, a total of 347 off-target genes were identified from microarray analysis (Birmingham et al., 2006). Surprisingly, the number of off-targets predicted using *in silico* analysis typically exceeded the number identified by microarray by 1–2 orders of magnitude. While these web-based bioinformatics searches can help to identify potential off-target genes, with the exception of the most obvious off-targets (those having identical or near-identical target sites), their effectiveness is limited due to their tendency to omit substantial numbers of functional siRNAs owing to unfounded specificity concerns (Birmingham et al., 2006). Additionally, identification of potential off-targets is limited by the large numbers of plant and other NTO’s genomes that remain to be sequenced and many that have been sequenced can have significant sequence variability due to mutations and recombination events within a species. Numerous studies have been conducted to better understand the effectiveness and specificity of individual siRNAs (and miRNAs). Many of these have looked at the effect of nucleotide mismatches on the efficacy of silencing of the target gene as well as for off-target gene silencing (Du et al., 2005; Dahlgren et al., 2008; Liu et al., 2014). Both single and double-nucleotide mismatches within the target gene were in many cases shown to still provide substantial levels of silencing (Du et al., 2005; Dahlgren et al., 2008). Both studies showed that the position as well as the identity of the mismatched base pair can have a significant effect on off-target silencing efficiency. These observations were advanced with the findings that base pairing in the seed-region (nt 2–7) of the 5′ end of the 21 base siRNA guide strand is the primary driving force of off-target activity and that there is also some involvement of the non-seed region (nt 9–20) in off-target silencing efficiency (Kamola et al., 2015). From their studies a number of parameters were recommended for minimizing off-target effects of highly functional siRNAs: (1) A or U at the 5′ end of the siRNA antisense (guide) strand; (2) G or C at the 5′ end of the siRNA sense (passenger) strand; (3) AU richness at the 5′ on-third region of the guide strand; (4) absence of any GC stretches more than 9 nt in length; (5) low T_m_ in the siRNA seed region; (6) high T_m_ in siRNA duplex/high GC content in the guide strand within the non-seed region (nt 8–15); and (7) high average GC content for target sequences corresponding to nt 8–15 of the guide strand. Similar seed and non-seed region effects were also noted for silencing by miRNAs (Broughton et al., 2016). The majority of these off-target studies were performed using artificial or specific human sRNAs introduced into cell cultures. Therefore, it remains to be determined if the OTEs (and recommended parameters to minimize these effects) demonstrated in animal systems will be applicable in plants and fungi. Undoubtedly, the parameters for design of siRNA for silencing efficiency and reduced potential for OTEs will be modified as additional studies are conducted in plant and fungal systems with more diverse arrays of siRNA targets as well as improved understanding of variability in the RNAi machinery of different target organisms.

## Movement of siRNA Between Host and Fungal Pathogen

The extent and longevity of down-regulation of fungal genes by host plant-induced RNAi depends on a number of factors including efficient uptake of siRNAs by the fungus, half-life of siRNAs, and if the siRNA signals can be amplified by the fungus. Several studies using fluorescently labeled siRNAs have shown significant uptake of siRNAs by fungi (Khatri and Rajam, 2007; Jochl et al., 2009). However, the exact mechanism by which exogenous RNAs enter into the fungal cell is not fully understood. Movement of RNA between plant host and invading pathogen represents an important phase of RNAi-mediated HIGS and while little is known about the mechanism of cross-species RNA transport, this aspect of HIGS is likely to be of importance in control of toxigenic fungi. Two major mechanisms related to host-derived RNA uptake by fungi have been postulated: (1) uptake of siRNAs via plant derived extracellular vesicles (EVs), and (2) active uptake via plasma membrane localized transporters.

### Vesicle Mediated RNA Transport

Vesicle mediated transport of macromolecules is reported to play critical roles in eukaryotes from the perspective of the host or the pathogen. In fungi, vesicle mediated transport of sRNAs has been described in several studies (reviewed by Haag et al., 2015). EVs of fungal origin are internalized by host cells either through endocytosis or intervention of extracellular fusogenic proteins (Knip et al., 2014). In mammals, RNA-sorting is shown to be an active process and mediated by membrane receptors. Preferential loading of sRNAs into EVs depends on factors such as size and the presence of specific nucleotide motifs in the 3′ UTR of the transcript and is regulated by the heterogeneous nuclear ribonucleoprotein (hnRNP) A2B1 in mammalian cells (Villarroya-Beltri et al., 2013). Several exogenous fusogenic proteins, e.g., syncitin and AFF-1 (Avinoam et al., 2011; Record, 2014), were shown to be involved in this process, though the exact mechanism is not fully understood. On the other hand, internalized exosomes are subjected to fusion with plasma membranes mediated by SNARE proteins (reviewed by Delic et al., 2013). The different aspects of RNA sorting and components (e.g., SNAREs) associated with exosome fusion to plasma membranes are equally pertinent to fungal pathogenicity and plant resistance as they are evolutionary conserved in both (Sansebastiano and Piro, 2014). In human pathogenic fungi including *Cryptococcus neoformans. Paracoccidioides brasiliensis. Candida albicans*, and also in the model fungus *Saccharomyces cerevisiae*, EV-mediated transfer of fungal RNAs (<250 nucleotide length) to human cells was identified and may possibly be involved in intercellular communication and pathogenesis (Peres da Silva et al., 2015). The abundant transcripts that were identified in the EV included, ASH1 (associated with cell budding), several heat shock proteins, fatty acid desaturase, glyoxylate pathway regulators, cytochrome b5 (CYB5), histone acetyl transferase (RTT109), cell division control protein CDC42 as well as other unique small non-coding RNAs enriched in the EV depending on the fungal species.

Though a plethora of information is available on vesicle-mediated RNA transfer in animal–pathogen interacting systems, there is a lack of direct evidence showing plant-derived vesicles delivering sRNAs to pathogenic fungi. Vesicle mediated transfer of plant sRNAs to fungi via an exosomal pathway is postulated based on the evidence gathered from several studies (Valadi et al., 2007; Nowara et al., 2010; Knip et al., 2014; Han and Luan, 2015). Vesicles released by plant cells are generally between 100 and 400 nm in diameter and are shown to be carriers of macro-molecules such as RNAs, proteins, and lipids (Ju et al., 2013; Mu et al., 2014). Several studies support the presence of exosome-like vesicles in plants and their role in delivering bioactive molecules such as sRNAs to animal cells (Mu et al., 2014; Raimondo et al., 2015). At this time it is not known if mechanisms other than vesicle-mediated transfer significantly contribute to the transfer of RNAs from plants to pathogenic fungi. If these specialized plant vesicles are the main mode of transfer and uptake of sRNAs from plant host to invading fungus, then the lifestyle of the fungus may play an important role in the efficacy of RNAi-based gene silencing. This may be especially relevant in case of necrotrophic fungi that destroy host cells during the course of colonizing the host plant. Degradation of host plant cells would likely have a negative impact on vesicle integrity leading to loss or severe reduction in the number of vesicles available for uptake by the invading fungus. Nevertheless, HIGS has been shown to be effective against necrotrophic plant pathogens but the actual mechanism of hpRNA/siRNA uptake by the pathogen from the host remains to be elucidated. Significant control of necrotrophs by plant-based RNAi approaches may in large part be dependent on sufficient uptake of sRNAs prior to the death of host cells and the presence of an efficient RNA-dependent RNA polymerase (RdRP)-mediated amplification of silencing signals by the fungal pathogen. It is clear that more research is needed in the areas pertaining to, (1) how sRNAs are loaded into plant vesicles inside the host (RNA-sorting?); (2) how sRNAs cross the host–fungus interface (i.e., are there fungal membrane receptors that bind to plant vesicles and internalize them?); and (3) if transfer is vesicle-mediated, how sRNAs compartmentalized in vesicles are released into the fungus?

### Transporter Mediated RNA Uptake

A few studies show involvement of transporters in RNA uptake in animal cells. In *Caenorhabditis elegans* the transmembrane protein SID-1, when expressed in *Drosophila* S2 cells (lacks a *sid-1* homolog) enables passive dsRNA uptake from the culture medium (Shih and Hunter, 2011). SID-1 can also transport dsRNA with single-stranded regions (hpRNA), pre-microRNA, and is involved in bi-directional dsRNA transport. Similarly, *C. elegans* apical intestinal membrane protein SID-2, when expressed in *Drosophila* S2 cells, facilitates dsRNA uptake via endocytosis (McEwan et al., 2012). The dsRNAs are released from internalized vesicles in a secondary step mediated by SID-1. In a recent study, Aizawa et al. (2016) identified a lysosome transmembrane protein SIDT2 in mammals, which is involved in RNA uptake (mRNA and rRNA) and subsequent degradation in the lysosome. At this time it is not known if similar dsRNA transporters exist in fungi but future studies in this area would provide important information on the relative contribution of transporter-mediated RNA uptake in RNAi silencing.

## Does RNA Size Influence RNA Uptake Efficiency?

Size dependent uptake efficiency of dsRNAs by the invading fungus is an important component of RNAi-based control approaches. In fungi, several studies have demonstrated active uptake of long and short dsRNAs resulting in silencing of target genes (Khatri and Rajam, 2007; Jochl et al., 2009; Kalleda et al., 2013). Disney et al. (2003) reported that both dsDNA and dsRNA are actively taken up by *C. albicans* cells, though higher uptake efficiencies were observed for linear nucleic acids vs. hairpin structures. Application of NaN_3_ (a metabolic inhibitor) to the fungal cells reduced dsRNA uptake by 10-fold suggesting presence of an active RNA transport system in *C. albicans*, but specific RNA uptake transporters in fungi have yet to be identified.

A size dependent uptake of dsRNA was reported in fruit fly (Saleh et al., 2006). In *Drosophila* S2 cell cultures long dsRNAs were more efficiently internalized than smaller RNAs. Based on luciferase reporter gene assays, exposure of flies with a 200 bp or greater dsRNA resulted in significant silencing of the reporter gene within an hour of incubation. Whereas, a 21 bp siRNA duplex had no effect on luciferase activity even after 30 h suggesting a preference for uptake of longer dsRNA vs. smaller and subsequent processing of these double stranded hpRNAs to generate silencing signals (siRNAs?). Similar results were observed in studies conducted in corn root worm (*Diabrotica virgifera virgifera* LeConte) (Bolognesi et al., 2012). Either oral application (through artificial diet) or incubation of midgut cells with dsRNA (Cy3-labeled) showed efficient internalization of 240 bp dsRNA and subsequent silencing of the target gene *Snf7*, a component of the ESCRT-III complex (endosomal sorting complex required for transport), whereas 21 bp siRNAs failed to enter into the cells. Silencing of *Snf7* resulted in growth inhibition and increased mortality of corn root worm. A size dependent efficacy of dsRNA-mediated silencing of the target gene was observed, as increased size of the dsRNA resulted in increased silencing. This observation could be due to the fact that larger dsRNAs result in production of a greater number of effective siRNA species targeting transcripts of the gene of interest. The size-dependent uptake of dsRNAs may be exclusive to worms and other higher eukaryotes. In fungi, both long and short dsRNAs are equally internalized and induce RNAi to silence target genes.

## Amplification of Silencing Signals

Effective RNAi relies on the signal-amplifying action of a specific RNA-dependent RNA polymerase (RdRP) capable of converting exogenously encountered dsRNAs into an abundant internal pool of secondary siRNAs (Pak et al., 2012). The presence of different paralogs of RdRPs in eukaryotes likely originated from gene duplication events and the paralogs are unique to distinct RNAi pathways. In plants, RNA-dependent RNA polymerase (*RdRp*) genes were found to play important roles in gene silencing and conferring resistance against invading pathogens. Inactivation of the rice *RdRp6* gene increased susceptibility to *Cucumber mosaic virus. Rice necrosis mosaic virus. Xanthomonas oryzae* pv. *oryzae* or *Magnaporthe oryzae* (Wagh et al., 2016). Small RNAs in plants can act as a systemic signal and travel long distances via phloem or plasmodesmata (from cell to cell) and affect gene expression (Molnar et al., 2010; Vatén et al., 2011). In *Arabidopsis*, gain-of-function mutant *cals3* exhibited increased accumulation of callose (β-1,3-glucan) at the plasmodesmata (PD) and decreased PD aperture. Higher accumulation of callose reduced intercellular sRNA trafficking resulting in shorter roots compared to the wild-type (Vatén et al., 2011). Even a low amount (10 ppm) of siRNA signal was sufficient to down-regulate reporter gene (GFP) expression at remote cells possibly due to the action of RdRP6 that amplified siRNA signals (Molnar et al., 2010). In another study, ingestion of dsRNAs (targeting vacuolar ATPase) supplied through artificial diet triggered RNAi in the coleopteran species, western corn rootworm (*D. virgifera virgifera* LeConte), which resulted in larval stunting and mortality. Considering the small amounts of dsRNAs required for gene silencing and larval mortality, the authors suggested a possible role of the amplification pathway in which ingested dsRNAs are processed to siRNAs, presumably within the insect gut epithelial cells, that might have primed the synthesis of more abundant secondary siRNAs (Baum et al., 2007).

Diverse modes of action of RdRP paralogs have been reported in fungi. In the zygomycete *Mucor circinelloides. rdrp-1* initiates silencing by sense transgenes through production of antisense RNA transcripts using the transgene, whereas *rdrp-2* efficiently amplifies the two different sizes of secondary siRNAs regardless of the nature of the trigger (Calo et al., 2012). Different modes of action of RdRPs are also evident in *Neurospora crassa* QDE-1 (QDE-1*^Ncr^*, RdRP component of the quelling pathway) and related fungi, *Thielavia terrestris* (QDE-1*^Tte^*) and *Myceliophthora thermophila* (QDE-1*^Mth^*) in synthesizing RNA. While QDE-1*^Ncr^* prefers processive RNA synthesis, QDE-1*^Tte^* and QDE-1*^Mth^* predominantly produce short RNA copies through a primer independent initiation process (Qian et al., 2016).

A similar amplification of silencing signals might also take place during the interaction of toxigenic fungal pathogens with plants harboring RNAi transgenes targeting fungal genes critical for growth and toxin production. In this scenario, plant-derived siRNAs targeting critical fungal gene transcripts can be amplified by fungal RdRPs upon uptake by the fungal cells and maintain sufficient threshold to down-regulate the targeted fungal genes (Masanga et al., 2015; Zhang et al., 2016).

## Half-Life of siRNAs

As sRNAs play critical roles in many biological processes in eukaryotes, any reduction or elevation of their levels in the cell can affect growth and development (Ji and Chen, 2012). Intracellular concentrations of sRNAs are controlled by their biogenesis and turnover rates. The half-life of sRNAs is increased by 2′-*O*-methylation on the 3′ terminal ribose of sRNAs. Small RNA methyltransferase, e.g., *HUA ENHANCER1* (*Hen1*) and its homologs are reported to methylate siRNAs and miRNAs in plants and flies (Horwich et al., 2007; Yu et al., 2010), and Piwi-interacting RNAs (piRNAs) in animals (Horwich et al., 2007; Saito et al., 2007). On the other hand, uridylation and 3′–5′ exonucleolytic degradation are attributed to the rapid turnover of sRNAs in plants and animals (Ji and Chen, 2012). *Arabidopsis hen1* mutants showed lack of methylation of siRNA and miRNA as compared to the wild-type counterpart (Yu et al., 2005). A reduction in abundance of miRNAs and size heterogeneity was also observed in the *Arabidopsis hen1* mutant. Reduction in abundance of miRNAs and trans-acting siRNAs were also observed in the rice mutant *waf1* (Abe et al., 2010), an ortholog of *Arabidopsis Hen1*. Suppressors of RNA-silencing by plant viruses, e.g., p19 (*Tomato bushy stunt virus* 19 kDa protein p19) were shown to interfere with sRNA methylation by *Hen1* (Lózsa et al., 2008). Several nucleotidyl transferases isolated from *C. elegans. Homo sapiens*, and *Chlamydomonas reinhardtii* were shown to either destabilize (by uridylation) or stabilize (by adenylation) sRNAs. Other factors affecting sRNA stability include the presence of specific *cis* elements (3′-terminal seven nucleotide sequence, ‘GGAUUCG’), that result in low stability of miR-382 in human cells (Bail et al., 2010). Components of RISC or RISC-associated factors were also shown to affect sRNA stability (reviewed by Ji and Chen, 2012). Argonaute proteins are shown to stabilize sRNAs by physical association or the slicer activity of the Ago proteins can also influence sRNA stability. Non-Ago proteins can also affect sRNA stability by stabilizing or destabilizing RISC. No information is available on RNA stability or rapid turn-over of sRNAs in fungi.

## Are siRNA Signals Transmitted Within the Plant and Also to Subsequent Generations?

Several studies have demonstrated systemic spread of siRNA signals in plants. In potato (*Solanum tuberosum* L.), foliar application of dsRNA against the Colorado potato beetle *actin* gene provided increased resistance against this pest and the resistance lasted for almost a month under greenhouse conditions (San Miguel and Scott, 2016). In tobacco (*Nicotiana tabacum* L. cv. Xanthi), foliar application of dsRNA targeting Tobacco mosaic virus (TMV) p126 (silencing suppressor) and coat protein genes, resulted in ∼50–65% resistance to this virus (Konakalla et al., 2016). The authors showed systemic spread of the silencing signal to the adjacent leaves within an hour and the presence of dsRNAs up to 9 days post-application. In a more recent study, Koch et al. (2016) showed that spray application of a long dsRNA (791 nt *CYP3*-dsRNA), which targets *Fusarium graminearum* (*Fg*) cytochrome P450, lanosterol, C-14α-demethylases genes (required for fungal ergosterol biosynthesis), significantly inhibited fungal growth both in the directly sprayed (local) as well as in the non-sprayed (distal) parts of detached leaves. Efficient spray-induced control of fungal infections in the distal tissue involved transport of *CYP3*-dsRNA via the plant vascular system and processing into siRNAs by *FgDCL-1* after uptake by the fungi. Taken into consideration, the above studies show the feasibility of foliar dsRNA application, subsequent uptake and processing of dsRNA, and systemic spread of the silencing signals in plants resulting increased disease resistance.

*Arabidopsis thaliana* (L.) Heynh. and tomato [*Solanum lycopersicum* (L.) Karst] plants challenged with caterpillar herbivory showed inheritance of resistance over two generations through a mechanism of DNA methylation (impacted by PolIV- and DCL2-dependent siRNA production) inherited through meiosis (Rasmann et al., 2012). Induced resistance was attributed to the transgenerational priming of jasmonic acid-dependent defense responses. *Arabidopsis* mutants defective in jasmonate perception (*coronatine insensitive1*) or siRNA biogenesis (*dicer-like2 dicer-like3 dicer-like4* and *nuclear RNA polymerase d2a nuclear RNA polymerase d2b*) failed to show inherited resistance. Similar transgenerational priming of a defense signaling pathway is reported against *Pseudomonas syringae* and *Hyaloperonospora arabidopsidis* in *Arabidopsis* (Luna et al., 2012; Slaughter et al., 2012).

If pathogens are once exposed to siRNAs (generated by transgenic RNAi plants), can this signal be perpetuated to subsequent generations of pathogens such that their ability to infect the host plant is significantly diminished? Perpetuation of siRNA signals negatively affecting fungal growth was observed when fungal spores isolated from infected RNAi plants were subcultured *in vitro* (Masanga et al., 2015; Zhang et al., 2016). There also exists the possibility in this scenario that the pathogen could evolve to overcome siRNA species to which they are chronically exposed. In this case, designing RNAi constructs that target a diverse range of genes critical for pathogenesis and mycotoxin production would be more meaningful toward achieving durable resistance. The perpetuation of siRNA signals to subsequent generations of a host plant that was exposed to a pathogen, or artificially applied siRNA through foliar application or seed priming, are being evaluated as next generation fungicides. Though not proven experimentally, the possibility exists that seeds could be primed with a mixture of synthetic siRNAs targeting an array of genes that are critical for pathogenesis and mycotoxin production in a wide variety of pathogens. The seed obtained from the plants derived from siRNA-primed seeds should have increased overall disease resistance and might be used for future crop production for a limited number of generations. This proposition is in line with the work earlier reported by Rasmann et al. (2012), where siRNAs were implicated in transgenerational disease resistance in plants. However, environmental factors may reduce the efficacy of transgenerational resistance based on the study of Zhong et al. (2013) showing suppression of PTGS by increased growth temperatures in *Arabidopsis*.

## Control of Fungal Pathogens by RNAi-Based Approaches

**FIGURE 2:**
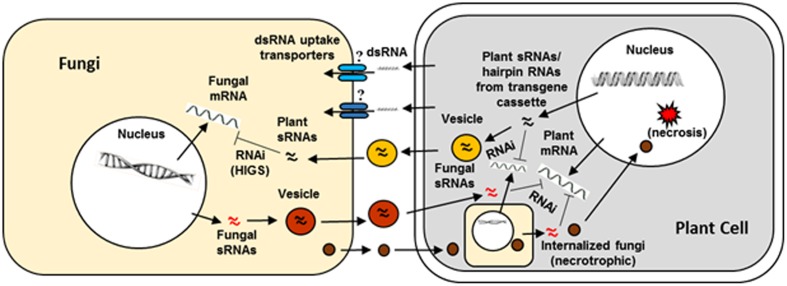
**Interaction between a plant cell and fungal pathogen in the context of plant RNAi- mediated host induced gene silencing.** Success of fungal colonization or plant resistance will depend on which organism, pathogen or host plant, is able to overcome the defense response of the other during the host–pathogen interaction. Plant small RNAs (sRNAs) produced as a consequence of normal defense response or from hairpin RNAs in transgenic RNAi plants (targeting a fungal gene) can cross plant and fungal barriers either through vesicles or RNA uptake transporters. The sRNAs after entering the fungi are released from the vesicles and initiate host-induced gene silencing. Similarly, fungal sRNAs can enter in to plant cells via vesicles and induce silencing of host defense genes. Besides sRNAs, effectors released by fungal cells enter into plant cells or the pathogen directly enters in to the host cell and repress host defense genes or activate host genes that are conducive for fungal growth eventually leading to necrosis.

There is a significant volume of literature on genes from major mycotoxigenic fungi such as *Aspergillus flavus. Fusarium*, and *Penicillium* species that play key roles in fungal growth, development, secondary metabolite production, virulence, and survival. These include genes encoding enzymes responsible for biosynthesis of toxic secondary metabolites as well as pathway-specific and global regulators of fungal secondary metabolism, development and stress response (reviewed in Amare and Keller, 2014; Qiu and Shi, 2014; Li et al., 2015). Success of earlier work using synthetic siRNAs to down-regulate key fungal genes involved in toxin production in *Aspergillus* and *Fusarium* indicated the feasibility of a hairpin RNA-based transgenic RNAi approach in plants to control mycotoxigenic fungi (McDonald et al., 2005; Abdel-Hadi et al., 2011). **Table 1** summarizes some examples of successful application of HIGS through transgenic RNAi-based approaches in crop plants or model systems targeting mycotoxigenic fungi. **Figure 2** elucidates a possible mechanism of RNAi-mediated silencing of fungal genes during the plant-fungus pathogenic interaction.

**Table 1:** Examples of application of host induced silencing (through RNAi) of true fungi and oomycete genes that are critical for growth and mycotoxin production.

Host plant	Pathogen	Target gene(s)	siRNA detection	Comments	Reference
*Zea mays*	*Aspergillus flavus*	*AflR*	NA	Reduced *AflR* gene expression as observed through semi-quantitative RT_PCR; a 14-fold reduction in aflatoxin contents (vs. control) in the kernels derived from RNAi lines; stunting and reduced kernel placement phenotypes of transgenic plants	Masanga et al., 2015
*Arachis hypogaea*	*Aspergillus flavus*	*aflS. aflR. aflC. pes1. aflep*	NA	∼4–20 fold expression of the hairpin RNA expression in the RNAi lines; ∼100% reduction in aflatoxin B1 and B2 in the seeds	Arias et al., 2015
*Hordeum vulgare*	*Fusarium graminearum*	*CYP51A. CYP51B*, and *CYP51C*	Northern blot	77–92% reduction in target gene expression; complete reduction of fungal growth with no disease symptoms in the RNAi lines	Koch et al., 2013
*Musa* sp.	*Fusarium oxysporum* f. sp. *cubense* (Foc)	*Velvet* and *Fusarium transcription factor 1*	RNAseq (∼0.9–8% of total RNAseq reads) through DIG-labeled probes	7–25 fold reduction in conidiophores count; increased resistance (70–85%) to *Fusarium* wilt	Ghag et al., 2014
*Triticum aestivum* L. (Yangmai15 cultivar)	*Fusarium graminearum*	*Chitin synthase (Chs) 3b*	Northern blot	1.4–4 fold reduction in *Chs 3b* expression; 78–85% reduction in deoxynivalenol (DON) contents	Cheng et al., 2015
*Arabidopsis thaliana* (Columbia 0)	*Fusarium oxysporum*	*F-box protein Required for Pathogenicity 1* (*FRP1*), *F. oxysporum Wilt 2* (*FOW2*), 12-*oxophytodienoate*-10,11-*reductase* gene (*OPR*)	NA	60–90% reduction in target genes expression; 15, 20–40, 35–60% increase in plant survival for *FOW2*-RNAi, *FRP1*-RNAi, and *OPR*-RNAi plants respectively. Significantly lesser number of yellow leaves in the RNAi plants	Hu et al., 2015
*Triticum aestivum*	*Fusarium culmorum*	β-1, 3-*glucan synthase* (Fc*Gls1*)	Semi-quantitative RT-PCR	Several fold reduction to complete silencing of Fc*Gls1* expression; 50–60% reduction in disease symptoms	Chen et al., 2016
*Gossypium* sp.	*Verticillium dahliae*	*Hygrophobins1* (*VdH1*)	RNA gel blot	Little to no signal of VdH1 gene expression from RNA gel blot analysis; ∼50–75% reduction in disease symptoms	Zhang et al., 2016
*Nicotiana tabacum*	*Phytophthora nicotianae. Peronospora tabacina*	*Cutinase*	RNA gel blot	Significant resistance against the pathogens in the RNAi plants, as evident from reduced foliar disease symptoms	Niblett and Bailey, 2012
*Glycine max*	*Phytophthora sojae*	*Cutinase*	RNA gel blot	Similar as described above	Niblett and Bailey, 2012
*Solanum tuberosum*	*Phytophthora infestans*	*Cutinase*	RNA gel blot	Similar as described above	Niblett and Bailey, 2012
*Triticum aestivum* cv. Thatcher	*Puccinia triticina*	*MAPK, cyclophilin* (*CYC1*), *and a calcineurin* (*CNB*) regulatory subunit gene	RNA blot hybridization	Increased resistance against the pathogen in the RNAi plants	Panwar et al., 2013


Transgenic corn (*Zea mays* L.) over-expressing hpRNAs targeting the aflatoxin gene cluster transcriptional activator gene *aflR*, showed significant reduction (14-fold vs. control plants) in aflatoxin content in kernels when challenged with an aflatoxigenic *A. flavus* strain (Masanga et al., 2015). Transgenic RNAi plants exhibited stunting and reduced kernel placement which the authors suggested may be the result of off-target effects of the siRNAs produced from the hpRNAs. In peanut (*Arachis hypogaea* L.), simultaneous silencing of five aflatoxin biosynthetic, transport, or non-ribosomal peptide synthetase (NRPS) related genes (*aflR. aflS. aflC. aflep*, and *pes1*) by RNAi resulted in 100% reduction in aflatoxin B_1_ and B_2_ content in transgenic plants compared to the control plants following inoculation with an aflatoxigenic *A. flavus* strain (Arias et al., 2015).

Successful implementation of HIGS has been reported against mycotoxin producing *Fusarium* spp., causal agents of head blight and root rot disease in cereals grown worldwide (Koch et al., 2013). HIGS of ergosterol biosynthetic genes of the *STEROL 14α-DEMETHYLASE* (*CYP51*) family in *F. graminearum* restricted fungal growth. A 791 bp dsRNA (*CYP3RNA*), complementary to *Fusarium CYP51A. CYP51B*, and *CYP51C* genes, inhibited fungal growth in *in vitro* feeding experiments. Fungal morphology showed a similar phenotype as observed when the same fungus is treated with the azole fungicide tebuconazole that targets *Fusarium* CYP51 enzymes. Transgenic plants of *Arabidopsis thaliana* and barley (*Hordeum vulgare* L.) over-expressing hpRNAs against *Fusarium CYP51* genes showed no fungal growth at the inoculation site. In banana (*Musa* sp.), RNAi-mediated silencing of the *Fusarium oxysporum* f. sp. *cubense velvet* and *transcription factor 1* genes, showed lack of external and internal infections in the transgenic lines in a 6-week-long (post-inoculation) greenhouse bioassay. Significant resistance (70–85% reduction in disease symptom) against *F. oxysporum* was observed in the RNAi plants at 8 months post-inoculation (Ghag et al., 2014). In *A. thaliana*, host-induced silencing of the *F. oxysporum* pathogenesis related genes, *F-box protein Required for Pathogenicity 1* (*FRP1*), *Wilt 2* (*FOW2*), and 12-oxophytodienoate-10,11-reductase (*OPR*), resulted in a 15–60% increase in plant survival depending upon the target gene silenced (Hu et al., 2015). RNA interference of the *F. graminearum* virulence gene, *chitin synthase* (*Chs. 3b*, resulted in a 74–76% reduction in disease symptoms in the spikelets of transgenic wheat (*Triticum aestivum* L. var. Yangmai15). A significant reduction (78–85%) in deoxynivalenol (DON) content was observed in the grains of RNAi plants under field conditions (Cheng et al., 2015). In another study conducted by Chen et al. (2016), transgenic wheat (*T. aestivum*) plants over-expressing hpRNAs against the *Fusarium culmorum* β-1, 3-*glucan synthase* gene (*FcGls1*) demonstrated enhanced *Fusarium* head blight resistance (∼50–75% reduction in disease symptoms) in leaf and spike inoculation assays under greenhouse and near-field conditions. Microscopic examination of *F. culmorum* colonies growing on *FcGls1*-RNAi plants revealed aberrant and swollen fungal hyphae with severe hyphal cell wall defects.

Effective RNAi against *Verticillium dahliae hygrophobin1* (*VdH1*) gene was reported to reduce disease symptoms by 50–75% in transgenic cotton (*Gossypium* sp.) over-expressing hpRNAs targeting this fungal gene (Zhang et al., 2016). Higher disease resistance in the transgenic cotton lines positively correlated with the presence of *VdH1* specific siRNAs and reduced expression of the target gene as evidenced from RNA gel blot analysis.

## Agricultural RNAi Risk Assessment

The major concern of transgenic RNAi-based approaches in the development of plants with improved agronomic traits is the potential for siRNAs generated by these plants (especially those destined for food and feed purposes) to have off-target effects (OTEs) (Petrick et al., 2013; Casacuberta et al., 2015; Roberts et al., 2015). Though not definitively proven, OTEs could adversely impact human and animal health due to gene suppression. There is also concern with respect to OTEs in plants that could result in adverse impacts on agronomic performance and crop quality. In 2014, the USA Environmental Protection Agency (EPA) convened a scientific advisory panel to address a number of questions with respect to potential impacts to human health and environmental risk assessment of pesticidal products using RNAi technology from which a report was issued (EPA-HQ-OPP-2013-0485-0049). In brief, the panel agreed that there is no convincing evidence that ingested dsRNA plant-incorporated protectants (PIPs) or naturally occurring plant miRNAs are absorbed from the mammalian gut in a form that causes physiologically relevant adverse effects. However, the panel recommended that the EPA (1) strive to collect additional data on dsRNA PIPs abundance and tissue distribution to evaluate factors that may affect absorption and effects of dietary dsRNAs; (2) conduct experimental testing of mammalian blood and exposed tissues to ensure that siRNAs processed from dsRNAs are not present that might lead to OTEs; (3) look into stability of different structural forms of dsRNAs to address the possibility of dermal or inhalation routes of exposure; and (4) investigate the stability of dsRNA in compromised individuals, the elderly and children. Questions raised with respect to OTEs of siRNAs in mammalian gut bacterial populations have been addressed. Unlike eukaryotes, bacteria lack the genetic components required for RNAi, but instead possess CRISPR/Cas systems where DNA is used as the gene silencing initiation signal (Horvath and Barrangou, 2010). Thus, it is questionable if dsRNAs could impact mammalian gut bacterial population considering the mechanistic differences between eukaryotic RNAi and CRISPR/Cas system in prokaryotes (Sherman et al., 2015).

The panel did find shortcomings in the EPA’s current biomolecule risk assessment approach with respect to ecological risks of dsRNA PIPs and concluded that additional data are needed to reduce uncertainty in environmental fate and ecological risk assessments. These include but were not limited to determination of environmentally relevant dosages of dsRNA PIPs, persistence of dsRNA in the environment, the importance of physical barriers in NTOs with respect to degradation and uptake of dsRNA, and OTEs to NTOs.

If transgenic plants expressing dsRNA PIPs are used for bioenergy purposes and not for consumption, or if the plant product goes through extreme industrial processing before consumption, then OTEs of siRNAs in mammals should not be a concern as siRNAs and naturally occurring miRNAs are likely to be degraded. While most risk assessments of plant RNAi-based PIPs have focused on mammalian, plant and arthropod-associated risks, no literature is available that describes potential risks to fungi. Risk assessments of plants engineered to express dsRNA PIPs targeting toxigenic fungal pathogens should also include possible adverse effects on non-target fungi inhabiting the plant rhizosphere, phyllosphere, and endosphere that play important roles in maintaining the health of the plant.

Besides OTEs, the efficacy of RNAi-mediated silencing of some target mRNAs can be complicated by copy number effects of the RNAi transgene. Integrated transgene cassettes can undergo transcriptional gene silencing due to multi-copy T-DNA integration at a locus adjacent to hypermethylated regions in the host genome (Kerschen et al., 2004). Concerns pertaining to RNAi induced methylation of homologous DNA including the RNAi transgene itself have also been raised (Casacuberta et al., 2015).

## Conclusion and Future Prospects

RNA interference has shown promise as a technology for control of fungal phytopathogens in food and feed crops as well as against a wide variety of other plant pests that result in loss of crop value. The fact that the mechanism of pathogen control by RNAi is not dependent on the plant’s need to produce a foreign protein that could be allergenic or toxic, should make this technology more acceptable than classic transgenic approaches currently used for disease control. In fact, if RNAi is used in conjunction with a precise genome editing tool to deliver an RNAi cassette to a desired location in the genome, disease resistant plants without any T-DNA backbone and possibly free from any selectable marker can be created. It would be unrealistic to expect complete disease or pest free RNAi-based transgenic plants, but any significant reduction in disease incidence can substantially reduce the application of toxic synthetic pesticides and would have a significant positive impact on agro-economy, human health, and ecosystem.

While concerns raised with respect to potential OTEs of RNAi to human and other mammal’s health appear to be unfounded, additional studies as put forth by the EPA advisory panel are needed to better determine the safety of transgenic dsRNA PIPs destined for consumption in food and feed products. Much more research is required to improve our understanding of the environmental fate of dsRNAs and their potential for uptake and induction of OTEs in NTOs. While new data is continuously being generated on the rational design of individual siRNAs to reduce potential OTEs, it remains difficult to accurately determine potential OTEs of transgenes expressing long dsRNAs that are capable of generating numerous siRNA species.

Transgenic RNAi is emerging as a powerful molecular tool for enhancing disease resistance traits in plants against a broad range of pests, including toxigenic fungi. Studies reported so far on the successful application of HIGS (RNAi) against fungal pathogens are mainly designed with the objective of pre-harvest control to reduce mycotoxin contamination in food and feed crops. To our knowledge there are no studies demonstrating efficacy of HIGS for control of mycotoxigenic fungi in a post-harvest storage scenario. It will be interesting to determine if RNAi-based approaches can combat post-harvest mycotoxin contamination as effectively as pre-harvest control. Theoretically, stored seed harvested from transgenic RNAi plants should offer some level of resistance against a target pathogen as the seed will contain both double- and single-stranded RNAs targeting the fungal gene(s) of interest. However, during post-harvest storage under low moisture conditions seeds are essentially dormant and therefore will not be able to maintain a steady production of hpRNAs/siRNAs. Stored double- or single-stranded RNAs present in transgenic RNAi seeds could still serve as an elicitor to initiate the RNAi pathway in the pathogen, but this needs to be experimentally validated. While RNAi-based control of toxigenic fungal plant pathogens is still in its infancy, it is clear that RNAi-based genetically modified plants are well on their way to commercialization (e.g., Monsanto’s SmartStax Pro for control of western corn earworm and DuPont Pioneer’s Plenish^®^ high oleic acid soybean). Initial studies on the efficacy of RNAi to control fungal pathogens have shown promise. However, these reports for the most part have been derived from laboratory and greenhouse studies, so durability and efficacy of this approach remains to be proven in field studies. It should also be taken into consideration that RNA silencing pathways appear to have diversified significantly in fungi because the numbers of RNA silencing proteins differ considerably among fungal species (Nakayashiki and Nguyen, 2008). This may lead to reduced efficacy of RNAi in some fungal species compared to others. Interestingly, in the causative agent of corn smut, *Ustilago maydis*, the entire RNA silencing machinery appears to have been lost, and thus should render this fungus insensitive to plant-based RNAi control approaches. Therefore, it will be important to determine by genome sequence analysis if in fact the target fungus has the full complement of RNAi machinery prior to initiation of transgenic plant-based RNAi studies.

Undoubtedly, due to the wide variation in the biology and physiology of plant pathogenic fungi and the plants they inflict disease upon, it will be impossible to adopt a “one size fits all” approach to RNAi-based control. This may be especially true when considering the lifestyle of the pathogen. Based on available literature, HIGS (RNAi) has been shown to significantly control (>50%) both biotrophic and necrotrophic plant pathogens. As biotrophs do not kill host cells, there should always be a steady supply of hpRNAs/siRNAs generated by the host plant for uptake by the fungus leading to efficient gene silencing. On the other hand, cell death caused by necrotrophs will rapidly deplete the source of hpRNAs/siRNAs leading to lower exposure of the fungus to these RNAs for uptake and subsequent gene silencing. Due to the potential of lower level exposure of a necrotroph to host plant generated hpRNAs/siRNAs, control may depend on the presence of an efficient RdRP-mediated amplification system in the fungus.

Each plant–fungal interaction will have to be addressed on an individual basis with emphasis placed on what key fungal gene(s) will be targeted for silencing and which promoter will be used to drive tissue- and developmental stage-specific expression of dsRNAs so as to best ensure uptake by the fungus. In addition, the success of RNAi-mediated control will depend in large part upon how well-researchers can address knowledge gaps in areas that impact efficacy and specificity of RNAi such as regulation of dsRNA and sRNA transport (host to pathogen and vice versa), fungal uptake of long dsRNA and sRNAs, prediction of OTEs, factors affecting sRNA stability in fungi, and amplification of the silencing signal. Bridging these knowledge gaps will enable scientists to confidently utilize RNAi technology in a highly efficient and specific manner to control mycotoxigenic fungi in susceptible crop plants.

## Author Contributions

RM and JC conceived the idea and prepared the draft of the manuscript. KR assisted with development of topics for discussion and edited the draft manuscript.

## Conflict of Interest Statement

The authors declare that the research was conducted in the absence of any commercial or financial relationships that could be construed as a potential conflict of interest.

## Footnotes

1 https://www.apsnet.org/edcenter/intropp/topics/Mycotoxins/Pages/EconomicImpact.aspx
